# Fatty Acid Biosynthesis Pathways Are Downregulated during Stigma Development and Are Critical during Self-Incompatible Responses in Ornamental Kale

**DOI:** 10.3390/ijms232113102

**Published:** 2022-10-28

**Authors:** Hongtao Qin, Hang Li, Kumar Abhinandan, Baoru Xun, Kun Yao, Jiayuan Shi, Ruoxi Zhao, Mugeng Li, Ying Wu, Xingguo Lan

**Affiliations:** 1 Key Laboratory of Saline-Alkali Vegetation Ecology Restoration, Ministry of Education, College of Life Sciences, Northeast Forestry University, Harbin 150040, China; 2 20/20 Seed Labs Inc., Nisku, AB T9E 7N5, Canada; 3 Department of Biological Sciences, University of Calgary, Calgary, AB T2N 1N4, Canada

**Keywords:** ornamental kale, stigma, self-incompatibility, flavonoid, fatty acid

## Abstract

In Brassicaceae, the papillary cells of the stigma are the primary site of the self-incompatibility (SI) responses. SI preserves the genetic diversity by selectively rejecting irrelevant or incompatible pollen, thus promoting cross fertilization and species fitness. Mechanisms that regulate SI responses in *Brassica* have been studied mainly on the mature stigma that often undermines how stigma papillary cells attain the state of SI during development. To understand this, we integrated PacBio SMRT-seq with Illumina RNA-seq to construct a de novo full-length transcriptomic database for different stages of stigma development in ornamental kale. A total of 48,800 non-redundant transcripts, 31,269 novel transcripts, 24,015 genes, 13,390 alternative splicing, 22,389 simple sequence repeats, 21,816 complete ORF sequences, and 4591 lncRNAs were identified and analyzed using PacBio SMRT-seq. The Illumina RNA-seq revealed 15,712 differentially expressed genes (DEGs) and 8619 transcription factors. The KEGG enrichment analysis of 4038 DEGs in the “incompatibility” group revealed that the flavonoid and fatty acid biosynthesis pathways were significantly enriched. The cluster and qRT-PCR analysis indicated that 11 and 14 candidate genes for the flavonoid and fatty acid biosynthesis pathways have the lowest expression levels at stigma maturation, respectively. To understand the physiological relevance of the downregulation of fatty acid biosynthesis pathways, we performed inhibitor feeding assays on the mature stigma. The compatible pollination response was drastically reduced when mature stigmas were pre-treated with a fatty acid synthase inhibitor. This finding suggested that fatty acid accumulation in the stigmas may be essential for compatible pollination and its downregulation during maturity must have evolved as a support module to discourage the mounting of self-incompatible pollen.

## 1. Introduction

Self-incompatibility (SI) in flowering plants is a genetic mechanism that promotes out-crossing while selectively rejecting self or incompatible pollen to maintain the genetic diversity of the population [1,2,3]. The stigma serves as the first “security checkpoint” during pollination [4,5] and pollen landing on the stigma triggers multi-level pollination related signal transduction pathways. If the pollen is compatible, a pollen tube penetrates through the cell wall of the papillary cells and travels down the transmitting tissue to the ovule, where it fertilizes the egg cells to complete the pollination process [6,7,8].

Successful pollination depends on a number of metabolic pathways and gene regulatory networks and healthy stigma development is critical for mediating the SI response [9,10,11,12,13]. The highly polymorphic *S*-locus contains SRK (female) and SCR/SP11 (male) determinants actively regulating SI [14,15,16,17,18]. As downstream signaling molecules, MLPK and ARC1 interact with SRK, actively regulating SI [19,20,21]. ARC1, an E3 ubiquitin ligase with U-box-dependent activity, ubiquitinates Exo70A1, GLO1, and PLD1 lead to SI response [22,23,24]. Interestingly, the C-terminus of JDP1 interacts specifically with ARC1 and Tyr8 at the N-terminus is identified as a specific site that regulates the interaction between JDP1 and ARC1 [25]. The SI response is also positively regulated by Ca^2+^ flux in stigma papillary cells. The application of SP11/SCR on papillary cell protoplasts increased Ca^2+^ flux while the GLR mutation led to a decrease in the Ca^2+^ concentration and interfered with the SI response [26]. Some candidate genes, such as THL1/THL2, directly interact with SRK to prevent its autophosphorylation and thus negatively regulate the SI response [27,28]. The GATA transcription factor *BnA5.ZML1* positively regulates the SI response by regulating the expression of SRK and ARC1 [29]. Interestingly, some metabolic factors have been shown to influence the SI response during pollination. ROS levels in the stigma affect pollen hydration and pollen tube growth. During the inhibition of RBOHs, a FER-Rac/Rop signal can reduce the level of ROS in the stigma, effectively breaking the SI [30]. The pollen-derived compatibility factor PCP-Bs competes with stigma-produced RALF23/33 for binding to the ‘stigmatic gatekeeper’ ANJ-FER complex, repressing the stigmatic ROS levels to facilitate pollen hydration [4].

In recent years, advanced DNA sequencing, proteome, and metabolic profiling technologies have helped researchers better understand the mechanisms and processes involved during stigma development and SI response. During SI in *Brassica napus*, 19 downregulated unique candidate proteins were identified using proteomic analysis of the stigmatic following incompatible pollination [31]. A total of 2500 mature stigma proteins were identified in *Brassica napus* using three different gel-based proteomics approaches, most of which participated in metabolic processes, responses to stimulus or stress, cellular, and developmental processes [32]. Two-dimensional difference gel electrophoresis (2D-DIGE) was used to identify 4000 protein spots and 107 proteins that were differentially expressed at five different stages of ornamental kale development [33]. Microarray technology and the cDNA subtraction library were used to identify 11,403 genes expressed in *Arabidopsis* stigma tissue and some stigma-specific and stigma-expressed genes were extracted [12]. *Brassica* gene expression microarrays were used to identify 287 stigmatic genes that are specifically regulated by compatible or self-incompatible pollinations in *Brassica napus* [34]. Laser microdissection and RNA-seq were used to identify 17,240, 19,260, and 21,026 cell type-specific genes in *Arabidopsis thaliana*, *A. halleri*, and *Brassica rapa* papilla cells, respectively [35]. The RNA-seq identified 293 highly expressed genes in *Brassica napus* stigma during compatible/incompatible pollen-stigma interactions [36].

With all the information generated using these technologies, we have only a few candidates and pathways that regulate the SI response. In addition, few transcriptional profiling studies have been performed on the different developmental stages of *Brassica* stigma. However, comparative transcriptome changes during different stages of development have not been performed. For the first time, we combined PacBio SMRT-seq and Illumina RNA-seq technology to investigate the comparative transcriptome of ornamental kale stigma at different developmental stages. We used small (SS, stage small), medium (SM, stage medium), and large (SL, stage large) stages of stigma to perform and analyze the full-length transcriptome using standard quality control methods. We confirmed the validity of the analysis using previously identified targets and reported a new pathway that significantly improves our current understanding of the SI response pathways. The results of this study will contribute to further understanding the related gene expression patterns and significant metabolic pathways in different stages of stigma development.

## 2. Results

### 2.1. Phenotypic Observation of Different Stages of Development and after Pollination in Stigma

**Figure 1:**
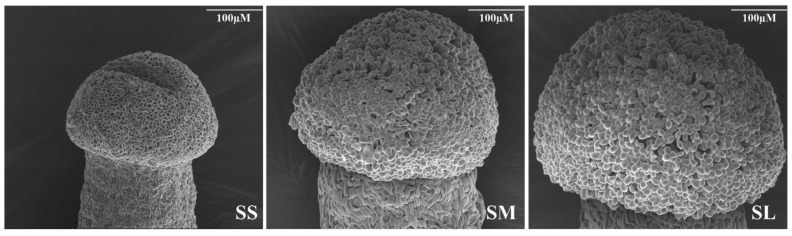
Morphological observation of different stages of stigma development. Stigma papilla cells were observed at different developmental stages using scanning electron microscope. Scale bars = 100 µM.

The morphological characteristics of stigmatic papilla cells at three different developmental stages were observed using a scanning electron microscope (Figure 1). Although the identity of the papillary cells did not establish yet, some cells have started to bulge and swell in SS stigma. The papilla cells have adopted a more defined oblate shape in SM stigma. The size of the papilla cells in SL stigma was significantly larger than SM stigma, and the cells appear nearly round and smooth at the top center and are tightly arranged.

**Figure 2:**
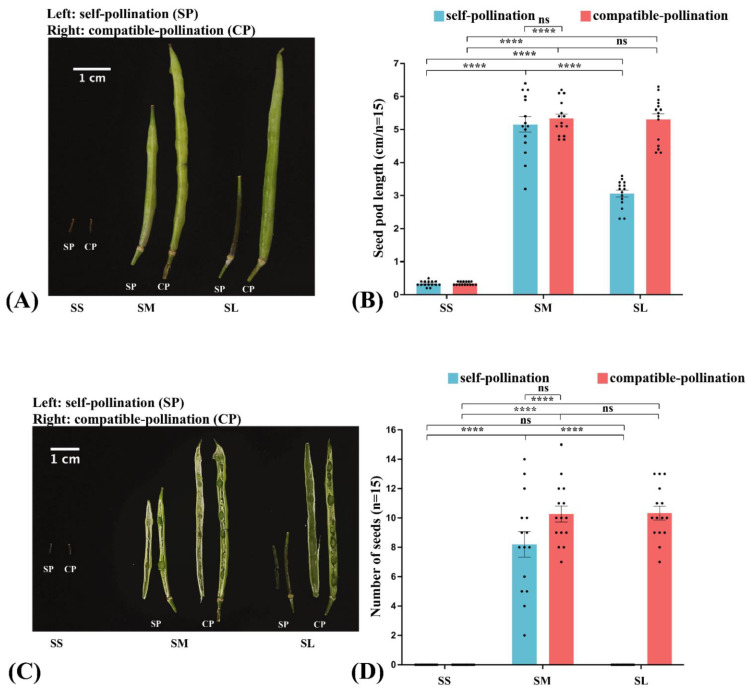
Observation of seed pod length and seed number at different stigma developmental stages after self-incompatible (SI) and compatible pollination (CP). (**A**) Seed pod morphology were observed after different pollination, (**left**): SI; (**right**): CP. (**B**) Seed pod length were counted after different pollination. (**C**) Seed morphology were observed after different pollination, **left**: SI; **right**: CP. (**D**) Seed numbers were counted after different pollinations. Asterisks indicate significant difference (**** *p* < 0.0001 by Student’s *t*-test), ns indicate no significant difference. Error bars represent the standard error of the mean (n = 15). Scale bars = 1 cm.

There are less pollen grain attachment and pollen tube growth in SL stigma after SI pollination. However, the number of pollen grains and tubes in SM stigma was significantly higher than in SL stigma (Figure S1A,B). The number of pollen grains and tubes in SM and SL stigma did not significantly differ after compatible pollination (CP) (Figure S1C,D). Both SI and CP showed a lot of pollen grain attachment and pollen tube growth at SM stigma. To further assess that SM stigma can accept SI pollen, the length of the seed pods and the number of seeds were measured and analyzed after SI and CP. The seed pods shriveled and could not develop into seeds at SL stigma after SI. The seed pods grew normally and developed to produce normal seeds after CP. However, all can grow seed pods and develop into seeds at SM stigma after SI and CP. Moreover, the length of seed pods and the number of seeds in SM stigma were significantly higher than in SL stigma after self-pollination (Figure 2A–D).

### 2.2. Full-Length Transcriptome Analysis Based on RNA-seq and SMRT-seq

**Figure 3:**
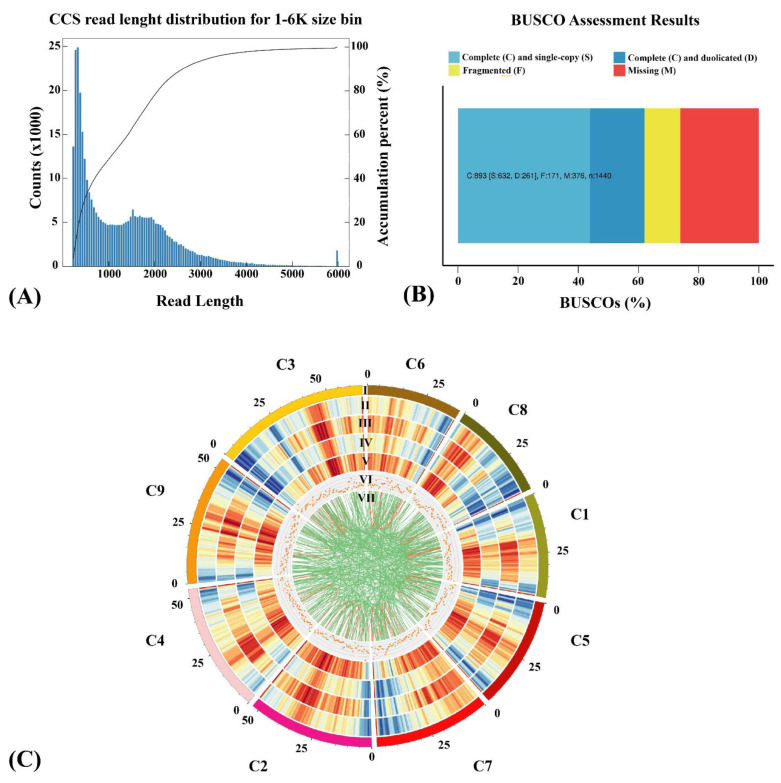
Full-length transcriptome quality assessment. (**A**) Distribution of CCS length. (**B**) BUSCO integrity assessment of full-length transcriptome data. (**C**) Circos visualization at the genome level. (I) Chromosome. (II) Gene density in the genome. (III) Gene density in PacBio SMRT-seq. (IV) Transcript density in the genome. (V) Transcript density in PacBio SMRT-seq. (VI) Distribution density of LncRNA on the chromosome. (VII) Distribution of the fusion transcript.

On the PacBio platform, 27.86 Gb of clean reads were obtained. After filtering adaptors and low-quality reads (less than 50 bp), 362,783 circular consensus sequencings (CCS) were generated with a mean read length of 1323 bp. CCS read lengths ranged from 0–6000 nucleotides (Figure 3A, Table S1). The CCS reads yielded 231,281 full-length non-chimeric (FLNCs), representing 63.75% of the total (Table S2). To eliminate up to 99% of sequencing errors, FLNC reads were clustered. There were 133,189 consensus isoforms with an average length of 1092 bp, including 126,165 polished high-quality (HQ) (94.73%; Table S3) and 4467 low-quality (LQ) isoform sequences. The LQ isoforms were then corrected using the Illumina RNA-seq data to enhance sequence accuracy. A total of 48,800 non-redundant transcripts and 24,015 genes were identified, of which 31,269 transcripts were novel; 62.0% of transcripts were found to be integrative, 43.9% were single copies, while 18.1% were duplicates. Missing or fragmented transcripts accounted for 38.0% of the total transcriptome (Figure 3B). The genome circos plot demonstrates that the density of genes and transcripts sequenced by the PacBio SMRT-seq platform is higher than that of the reference genome, while LncRNA and fusion transcript were evenly distributed on each chromosome (Figure 3C).

### 2.3. Identification of Fusion Transcript, AS, APA, SSR, ORFs, and lncRNA

**Figure 4:**
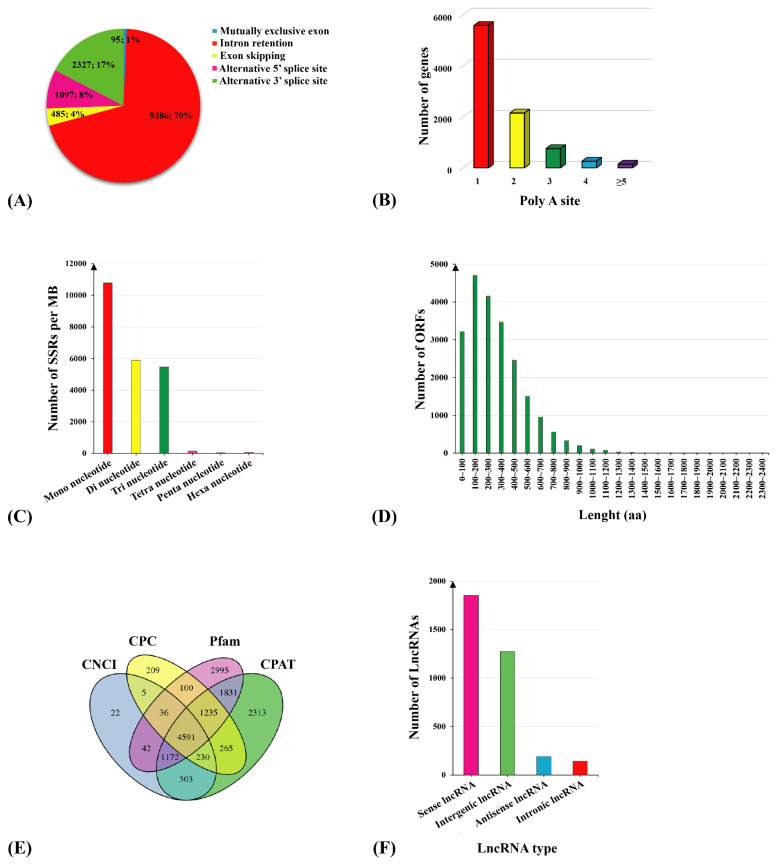
Prediction of AS, APA, SSR, ORF, and lncRNA events. (**A**) Number statistics of AS events. (**B**) Number statistics of the APA sites. (**C**) Density analysis of the SSR type. (**D**) Distribution map of the ORF–encoded protein length. (**E**) Venn diagram of LncRNA screening methods. (**F**) Classification map of the lncRNA location.

The PacBio-seq platform has the significant advantage of identifying gene alternative splicing (AS) events. A total of 13,390 AS events were found after AS analysis of 48,800 non-redundant transcripts, including 9386 (70%) intron retention (IR), 2327 (17%) alternative 3′ splice site (A3), 1097 (8%) alternative 5′ splice site (A5), 485 (4%) exon skipping (ES), and 95 (1%) mutually exclusive exon (MEE) (Figure 4A). Additionally, 8899 genes were found to contain alternative polyadenylation (APA) sites, of which 3315 (37.3%) genes had two or more polyA sites and 5584 (62.7%) genes contained only one polyA site (Figure 4B). A total of 22,389 Simple Sequence Repeats (SSRs) were detected. The SSR pool consisted of 10,779 mono, 5895 di, 5475 tri, 144 tetra, 37 penta, and 59 hexa nucleotides (Figure 4C). TransDecoder software was used to predict 21,816 complete open reading frames (ORFs) of novel transcripts and their corresponding amino acid sequence (Figure 4D). The coding potential calculator (CPC), the coding noncoding index (CNCI), the coding potential assessment tool (CPAT), and the PFAM software were used to identify 4591 long noncoding RNAs (lncRNAs), which included 1853 sense lncRNAs, 1274 intergenic lncRNAs, 191 antisense lncRNAs, and 141 intronic lncRNAs (Figure 4E,F).

### 2.4. Identification and Functional Analysis of TFs and DEGs

A total of 8619 transcription factors (TFs) were predicted and categorized into 216 TF families. Based on the gene count, the highest represented TF families were further analyzed (Figure S2A). The bHLH family accounted for the largest share (332 genes), followed by the RLK-Pelle DLSV family (322 genes), the AP2/ERF-ERF family (279 genes), and the MYB family (277 genes). WGCNA was performed based on the expression profile of 8619 TFs in three stages of stigma development to construct gene co-expression modules and investigate the gene regulatory network of the various stages of stigma development. The three distinct stages of stigma produced 12 highly correlated TF modules (Figure S2B,C).

The DEGs were classified into three comparison groups to investigate the gene expression pattern of the stigma at different developmental stages, including SS vs. SM, SS vs. SL, and SM vs. SL. The three groups yielded 15,712 DEGs, of which 6349 (3063 upregulated and 3286 downregulated) DEGs were found in the SS vs. SL comparison group, accounting for majority of them. In the comparison groups, for SM vs. SL and SS vs. SM, respectively, 5185 (2870 up-regulated genes and 2315 downregulated genes) and 4178 (2166 up-regulated genes and 2012 downregulated genes) DEG were discovered (Figure S2D–F; Table 1).

DEGs in three comparison groups were annotated using the seven databases, of which 5837 DEGs in COG, 14,810 DEGs in eggNOG, 15,170 DEGs in NR, 12,566 DEGs in Pfam, 11,714 DEGs in Swiss-Prot, 14,272 DEGs in GO, and 5304 DEGs in KEGG (Table S4). According to the results of the GO enrichment analysis, the 3775 (SS vs. SM), 5828 (SS vs. SL), and 4669 (SM vs. SL) DEGs were divided into 52 classes, with the majority of DEGs falling into one of three broad categories: (metabolic process, cellular process, and biological process). The most enriched terms for molecular function were binding, catalytic activity, and nucleic acid binding transcription factor activity (Figure S3A–C). In each of the three comparison groups, 20 significant KEGG enrichment pathways are shown in Figure S3D–F. A Venn diagram was used to further analyze the distinct and overlapping sets of DEGs. In the three comparison groups, 717 common DEGs that may be essential to the metabolism process were found at the various stages of stigma. There were 1691 specific DEGs between SS and SM, 1249 between SS and SL, and 995 between SM and SL, indicating that the difference between SS and SM was the greatest.

**Figure 5:**
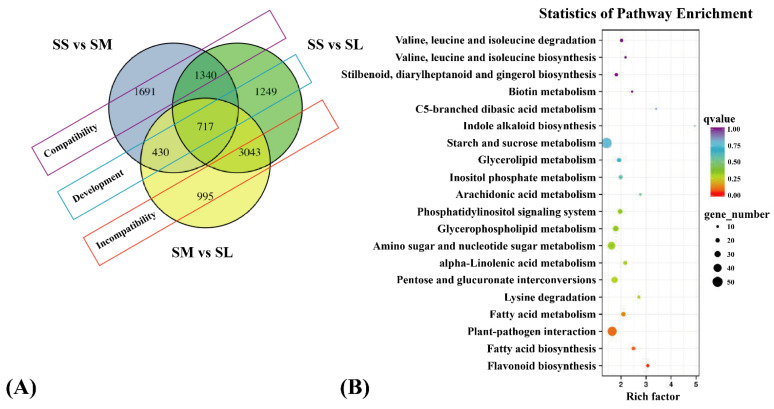
Functional division and KEGG analysis of DEGs. (**A**) Functional division of DEGs in the three comparison groups. The purple wire frame represents the differential gene in the compatibility groupt; the blue wire frame represents the differential gene in the developmental group; the red wire frame represents the differential gene in the “incompatibility” group. (**B**) KEGG enrichment analysis of DEGs in the “incompatibility” group.

The data was used to create a Venn diagram of DEGs, which was then used to categorize the DEGs into three groups: “compatibility”, “development”, and “incompatibility” (Figure 5A, Table S5). To further explore the biological metabolic processes involved in DEG during the “incompatibility” group, the 4038 DEG of the “incompatibility” group were annotated using the KEGG database. The flavonoid and fatty acid biosynthesis pathways were significantly enriched among the 20 KEGG enrichment pathways (Figure 5B). In order to understand effective SI regulatory networks, it is necessary to investigate the genes that play a role in the metabolic process involving flavonoids and fatty acids.

### 2.5. Cluster Analysis and qRT-PCR Validation of Genes Related in the Flavonoid Biosynthesis Pathway

**Figure 6:**
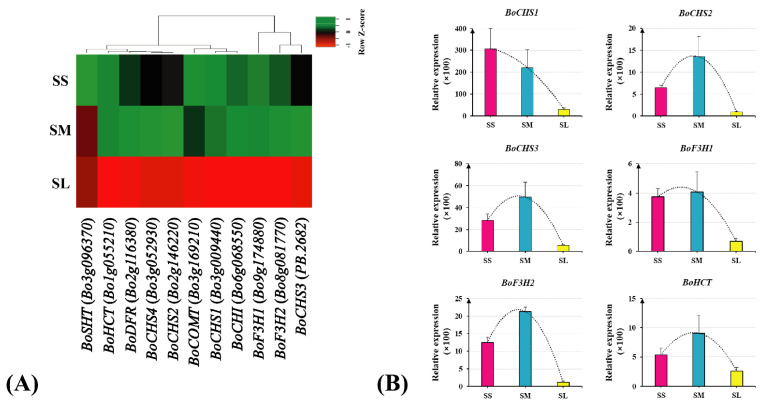
Cluster analysis and qRT−PCR validation of candidate genes related in the flavonoid biosynthesis pathway. (**A**) Heatmap of candidate gene expression profile. (**B**) qRT−PCR analysis of candidate genes related to the flavonoid biosynthesis pathway.

The KEGG analysis indicated that flavonoid and fatty acid biosynthesis pathways were significantly enriched. The heat map of 11 flavonoid biosynthesis genes was shown based on the quantitative results of the transcriptome analysis data. The expression levels of these key genes involved in flavonoid biosynthesis showed downregulation in the SL stigmas (Figure 6A). To validate the reliability of the transcriptome analysis data, six key enzyme genes for flavonoid biosynthesis were selected for qRT-PCR analysis (Figure 6B). The results showed that RNA-seq and qRT-PCR revealed high-ranking consistency, indicating that the RNA-seq data for this study is accurate and effective. The expression levels of six key enzyme genes involved in flavonoid biosynthesis showed a downregulation trend during the stigma SS vs. SL and SM vs. SL stigmas. Except for *BoCHS1*, *BoCOMT* and *BoSHT*, all other genes showed a peak in SM stigma and a drastic reduction in SL stigma.

### 2.6. Functional Analysis of Related Genes in the Fatty Acid Biosynthesis Pathway

**Figure 7:**
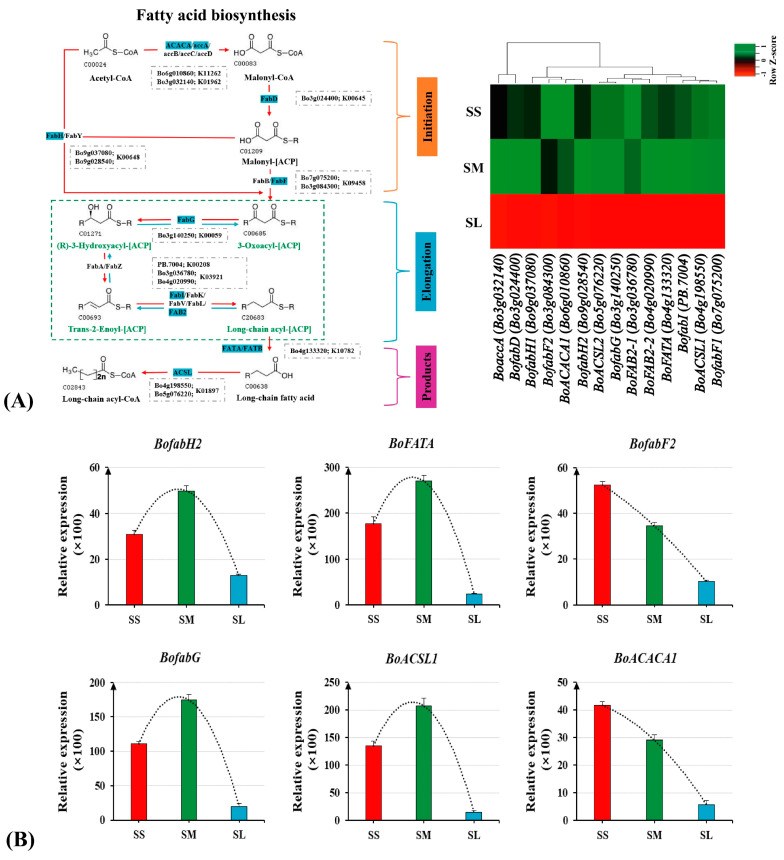
Cluster analysis and qRT−PCR validation of candidate genes related in the fatty acid biosynthesis pathway. (**A**) Fatty acid biosynthesis pathway map and heatmap of candidate gene expression profile. (**B**) qRT−PCR analysis of candidate genes related to the fatty acid biosynthesis pathway.

In the fatty acid biosynthesis pathway, 14 important enzyme genes were investigated. Figure 7A illustrates the precise regulatory mechanism involved in the synthesis of fatty acids. A heatmap of 14 fatty acid biosynthetic genes were generated based on the quantitative results of transcriptome analysis data. The expression levels of these key genes involved in fatty acid biosynthesis showed a downregulation in the SL stigmas (Figure 7A). Six key enzyme genes for fatty acid biosynthesis were selected for qRT-PCR analysis, and RNA-seq and qRT-PCR revealed high-rank consistency (Figure 7B). The expression levels of six key enzyme genes involved in flavonoid and fatty acid biosynthesis showed a downregulation trend during the stigma SS vs. SL and SM vs. SL stigmas. Except for *BofabF2* and *BoACACA1*, all other genes showed a peak in SM stigma and a drastic reduction in SL stigma.

**Figure 8:**
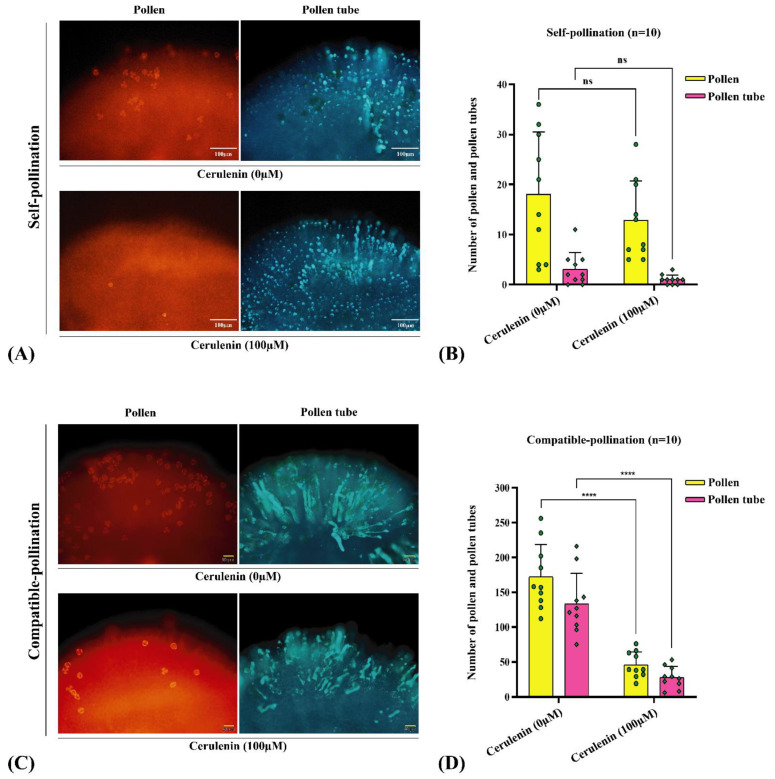
Effects of the fatty acid synthase (FASN) inhibitor on self-pollination and compatible pollination. (**A**) Fluorescence images of self-pollination after stigma were treated with cerulenin (100 µM). (**B**) Pollen and pollen tube number statistics of self-pollination after stigma were treated with cerulenin (100 µM). (**C**) Fluorescence images of compatible pollination after stigma were treated with cerulenin (100 µM). (**D**) Pollen and pollen tube number statistics of compatible pollination after stigma were treated with cerulenin (100 µM). Asterisks indicate significant difference (**** *p* < 0.0001 by Student’s *t*-test), ns indicate no significant difference. Error bars represent the standard error of the mean (n = 10). Scale bars = 50/100 µM.

To investigate the role of fatty acids during pollen-stigma interactions, we further investigated the effects of the in vitro application of cerulenin [a potent natural inhibitor of fatty acid synthase (FASN) inhibitor] on pollination. The SL stigmas from the self-incompatible ornamental kale line (*S_13-b_S_13-b_*) were pretreated with cerulenin and the effects on self and cross-pollination for pollen attachment and pollen tube formation were observed. Compared to untreated stigmas, the results established that the stigma feeding assay with FASN inhibitor (cerulenin) did not alter the number of pollen grain attachments and pollen tube growth after self-pollination (Figure 8A,B), while drastically reducing compatible pollen grain attachment and pollen tube growth after CP (Figure 8C,D). Thus, fatty acid accumulation in stigmas is essential for compatible pollinations. Therefore, it seems likely that Brassicaceae species evolved to block the accumulation of fatty acids in mature stigmas since this could abrogate the SI response.

## 3. Discussion

Currently, several studies have only identified differentially expressed genes and proteins during the pollination process during stigma maturity [12,31,32,34,35,36]. Few comparative transcriptome analyses of different stages of development in stigma have also been reported. PacBio SMRT-Seq helps in uninterrupted long RNA reads that contain a single complete transcript sequence information, and no assembly is required for later analysis. PacBio SMRT-Seq has obvious advantages in the mean length of unigenes and the ratio of full-length transcripts compared to Illumina RNA-Seq [37,38]. PacBio SMRT-Seq combined with Illumina RNA-Seq was used to generate a comprehensive transcriptome. There was a total of 48,800 non-redundant transcripts, of which 31,269 novel transcripts were discovered; 22,389 SSRs, 21,816 ORFs, and 4591 lncRNAs were determined. The precursor mRNA was produced using gene transcription and had many splicing mechanisms, and different exons were selected to produce different mature mRNA, thus translating into different proteins, constituting the diversity of biological traits [39]. PacBio SMRT-seq has the advantage of effectively and accurately identifying AS events. PacBio SMRT-seq discovered 12,293 AS events, providing new insights for future studies on gene expression regulation and protein diversity. Illumina RNA-seq revealed 15,712 DEGs (8099 upregulated and 7613 downregulated) and 8619 TFs in three comparison groups. WGCNA was performed to reveal the modules of highly correlated TFs [40], and 12 highly correlated TF modules were obtained that could play essential roles in different developmental stages. The enrichment analysis of the KEGG pathway showed that the DEGs of the “incompatible” group were significantly enriched in the flavonoid and fatty acid biosynthesis pathways that contained 11 and 14 candidate genes, respectively. This information can help in the discovery of critical regulatory network of the SI genes and the metabolism pathway during stigma development.

In *Arabidopsis*, flavonoid levels impact plant root growth, seed development and germination, and pollen development, release and vitality, and flavonoids are necessary for full fertility [41]. Flavonoids have been found to accumulate in a variety of tissue types, including male and female sex organs, which play an important role in sexual reproduction [42,43]. Chalcone synthase (CHS), chalcone isomerase (CHI), flavanone 3β-hydroxylase (F3H), flavanol synthase (FLS), and dihydroflavonol 4-reduc-tase (DFR) are key enzyme genes in the pathway of flavonoid metabolism, which play an important regulatory role in the accumulation of flavonoids [44,45]. Flavonoid levels in stigmas play an important role in regulating the completion of pollination events. Lan et al. [33] indicated that flavonoids could act as antioxidants to break SI, four flavonoid enzymes (CHS1, CHS2, F3H, and FLS) were identified; additionally, a dramatic reduction during the ornamental kale stigma maturation stage to prevent any interference with the SI response, concomitant with an increase in ROS, implied that there is an interaction between flavonoids and ROS. However, this study determined 11 key enzyme genes for flavonoid biosynthesis, which also showed a reduced trend in the stage of stigma maturation, and further enriched the number and variety. Zhang et al. [30] found that ROS levels in the stigma of Chinese cabbage had a strong effect on pollen germination and pollen tube growth. ROS levels in the stigma increased after self-pollination and ROS levels in the stigma decreased after compatible pollination, indicating that higher levels of ROS in the stigma can lead to SI responses.

Lipids provide a nutrient rich environment for pollen hydration and germination in *Brassica napus* [46,47]. Fatty acids are common components of lipids. In this study, several genes encoding fatty acid key enzymes were uncovered, providing potential candidate genes for future research into the effect of fatty acid biosynthesis on pollination and related mechanisms. Overall, fatty acid regulation and metabolism plays a major role in the growth and development of living organisms. For example, ACSL acts on a broad range of long-chain saturated and unsaturated fatty acids and fadD catalyzes the formation of malonyl-ACP, which serves as an elongation substrate in fatty acid biosynthesis. *ACSL* genes were up-regulated in a salt-stressed oleaginous diatom in response to salt stress [48]. Soybean *GmACSL2* overexpression in the hairy roots of soybean severely reduces the content of lipids and fatty acids and may participate in promoting seed germination [49]. A reduced expression of FATA thioesterases can reduce the oil content and fatty acid composition of the seed in *Arabidopsis* [50]. The heterologous expression of *Jatropha curcas JcFATA* and *JcFATB* in *Arabidopsis thaliana* can promote plant growth and development (longer roots, stems and siliques, larger rosette leaves, and bigger seeds), with its facilitative effects on the increase in the fatty acids content [51]. Fatty acid elongase is required for shoot development in rice [52]. Fatty acid desaturases (FADs) can improve plant resistance by modulating lipid metabolism, stability and fluidity of cell membranes, reactive oxygen species signaling pathways, etc. [53].

The present study confirms that SM stigma is capable of supporting pollen germination and fertilization, however this stage corresponds to before anthesis when the stigma is still naturally unexposed to the environment. Unless manually supported, even self-pollination during SM is highly unlikely since the anthers mature simultaneously during anthesis and pollen grains are released once the anther is mature. It has been shown that the expression of SRK is at peak during anthesis and the SM stigma is only sparsely localized by the SRK responsible for receptor ligand interaction. Thus, the confounding effects due to such regulation posed by the presence of SRK might cause confusion towards the role of other candidate pathway genes during SI.

The SM stigma had the ability to recognize and accept both self-pollination and compatible pollination, the SM stigmas can temporarily break the SI response and produce fertile offspring. It is speculated that the fact they can temporarily break the SI phenomenon exhibited in SM stigma is closely related to the increase in the expression level of key enzyme genes for flavonoid and fatty acid; this may be potentially related to the accumulation of fatty acid and flavonoid contents in the SM stigma that can interfere with self-incompatibility. The qRT-PCR and cluster analysis indicated a significant reduction in the flavonoid and fatty acid biosynthesis pathways during stigma development, and it seems likely that Brassicaceae species evolved to block the accumulation of flavonoid and fatty acids in mature stigmas. Therefore, further research is required to determine whether fatty acids and flavonoids have a dependent and synergistic relationship in response to SI.

This is the first study to suggest that these fatty acid biosynthesis genes play a crucial role in pollination. We used cerulenin that covalently binds to the catalytic site of FAS and disrupts the condensation reaction of acetyl-COA and malonyl-COA, inhibiting fatty acid biosynthesis [54,55,56]. It drastically reduced the compatible pollen attachment and germination in SL stigma, indicating that the upregulation of fatty acid pathways during stigma maturity might promote the germination of unsuitable/undesirable pollen grains, which might lead to a waste of maternal resources and lead to unfit off-springs.

## 4. Materials and Methods

### 4.1. Plant Materials

The ornamental kale (*Brassica oleracea* var. *acephala*) self-incompatible *S_13-b_S_13-b_* and compatible *S_45_S_45_* line were grown in the flower bioengineering institute of the Northeast Forestry University. The self-incompatible *S_13-b_S_13-b_* stigmas were classified into three different stages of development based on the length of the floral bud length [(SS = 0–4 mm, stage small), (SM = 6–8 mm, stage medium), (SL ≥ 10 mm, stage large)] [33]. The morphological changes of the stigma at different developmental stages were observed using the scanning electron microscope.

### 4.2. Total RNA Extraction

The stigmas of different developmental stages in the *S_13-b_S_13-b_* line were set up with three biological replicates, 300–500 stigmas were collected for each replicate and placed in 1.5 mL of RNase-free EP tubes and immediately frozen in liquid nitrogen; the sample is stored at −80 °C. The total RNA was extracted from 9 samples of three stages of stigma development using the RNA preparation kit (Tiangen). The quality of purified RNA was initially evaluated on 1.5% agarose gel and then the RNA concentration was measured using NanoDrop 2000 (Thermo Scientific, Waltham, MA, USA).

### 4.3. PacBio SMRT-Seq and Illumina RNA-seq Library Preparation and Sequencing

A mixed RNA of the 9 samples in three stages of stigma development was used to construct the Illumina and PacBio cDNA library. Full-length cDNA was synthesized using the Clontech SMARter PCR cDNA Synthesis Kit and filtered using BluePippin. The PCR was used after the optimization cycle to generate double-stranded cDNA on a large scale, the BluePippin™ Size Selection System (Sage Science, Beverly, MA, USA) was used to perform size fractionation; full-length cDNAs were performed, DNA damage repaired, end repaired, ligated to sequencing adapters, and then digested with exonuclease; the qualified cDNA libraries were sequenced using the PacBio sequel platform (Pacific Biosciences, Menlo Park, CA, USA). Eukaryotic mRNA was enriched by magnetic beads with Oligo (dT); Fragmentation Buffer was added to randomly interrupt the mRNA; using mRNA as template, the first cDNA strand was synthesized with six random primers; DNA polymerase I and RNase H were used to synthesize the second strand cDNA; the cDNA was purified using AMPure XP beads; end repair of purified cDNA was performed; and, after adding A-tail and connecting the sequencing adapter, the cDNA libraries were enriched using PCR. Finally, the qualified cDNA libraries were sequenced using the Illumina platform.

### 4.4. PacBio SMRT-seq and Illumina RNA-seq Data Processing

A SMRT cell with a cDNA size of 1–6K was sequenced. The raw data from PacBio Sequel were processed using the SMRTlink 6.0 software. CCSs were generated from effective subreads’ BAM file with full passes ≥3, accuracy ≥0.9. The CCS reads were classified into FLNC, non-full length according to whether there were 5′ and 3′-cDNA primers, and poly(A) tail signal. The full-length reads were clustered using ICE software and further polished to generate HQ consensus isoforms. The full-length transcripts of the HQ (accuracy ≥ 0.99) and LQ were identified using the Quiver algorithm. Finally, non-redundant HQ full-length transcripts were obtained using the program CD-HIT. Raw reads were obtained using the high-throughput Illumina sequencing platform, and clean reads were obtained by removing the raw reads containing adapters, poly-N, and LQ reads. The clean reads were then mapped back to the assembled full-length transcripts of SMRT-Seq. Sequences with identities less than 0.90 and coverage less than 0.85 were filtered out. The HQ non-redundant transcript sequences were obtained using cDNA_Cupcake software. The integrity of the transcriptome was assessed using the universal single-copy benchmark orthologs (BUSCO).

### 4.5. Prediction of Fusion Transcript, AS, APA, SSR, ORFs, and lncRNAs

The fusion transcript of the entire transcriptome was screened using the following criteria: (a) mapping to two or more loci; (b) each locus must be covered with at least 5% of the transcript length; (c) total coverage length must account for more than 95% of the total length of the transcript; (d) the distance between adjacent loci was 10 kb. Transcripts that contained complete ORF, 5′ and 3′-UTR, were designated full-length transcripts. AS events include IR, ES, AD, AA, and MEE; AS events of all non-redundant full-length transcripts were detected using the ASTALAVISTA program, the Cuffdiff tool was used to identify these AS events using transcript models obtained from cufflinks. The FLNCs were further analyzed to identify APAs through the TAPIS pipeline. The SSR analysis of the entire transcriptome was performed using the MIcroSAtellite Identification Tool (MISA). The seven types of SSRs were identified by analyzing transcript sequences, including mononucleotide, dinucleotide, trinucleotide, tetranucleotide, pentanucleotide, hexanucleotide, and compound SSR. The ORFs in the transcripts were predicted to define putative coding sequences (CDS) using the package TransDecoder. The candidate lncRNAs were predicted based on CNCI, CPC, CPAT, and PFAM.

### 4.6. Identification and Functional Annotation of DEGs

Clean reads were mapped against the assembled reference full-length transcripts using Bowtie2 software. Gene expression levels in different stages of stigma development were calculated using RSEM software and read counts were normalized using fragment per kilobase of transcript per million fragments mapped (FPKM). The DESeq R package (version 1.10.1) was used to screen significant DEGs (FPKM > 10, FDR < 0.01, and |log2 (Fold Change)| ≥ 1). Finally, the TFs were predicted using iTAK software. The functional annotations of the DEGs were performed using BLAST software against 8 different databases; the relevant analysis databases are shown in (Table S6). Biosynthetic metabolism pathways and signal transduction pathways of different developmental stages of stigma in ornamental kale were screened by searching for results of integrative annotation.

### 4.7. Construction of WGCNA

Weighted gene co-expression network analysis (WGCNA) of TFs at stigma different developmental stages was performed via the BioMarker cloud platform. FPKM values of the transcripts from the 9 samples belonging to the three developmental stages in stigma were used for WGCNA. Different branches of the clustering tree represent different gene modules, the modules were defined as highly interrelated gene clusters, and the genes in the same cluster showed high correlation coefficients. The gene regulatory network was drawn using the OmicShare tools and the genes highly related to different developmental stages of stigma were identified.

### 4.8. qRT-PCR Analysis

Total RNAs were obtained from the previous samples used for sequencing. The cDNA was synthesized with the HiScript^®^Q RT SuperMix for qPCR (Vazyme). RT-qPCR was performed using SYBR qPCR Mix (TransGen) on the LightCycler480 System (Roche). The relative expressions of all tested candidate genes were normalized to the internal control ACTIN. Three biological replicates were performed for each RT-qPCR experiment. The relative gene expression level was calculated by 2^−∆∆CT^ method. RT-qPCR primers were designed with Primer Premier 5.0 and the primer sequence used in this experiment is listed in Table S7.

### 4.9. Pollination Experiment

The *S_13-b_S_13-b_* self-incompatible line selected for emasculation, self-pollination (*S_13-b_S_13-b_* pollen), and compatible-pollination (*S_45_S_45_* pollen) were carried out. The aniline blue staining method was provided by Lan et al. [33]; using it, the pollen grain attachment, pollen tube growth, seed pod lengths, and seed numbers were observed after self-pollination and compatible pollination.

### 4.10. FASN Inhibitor Treatment and Pollination Visualization

Ornamental kale (*S_13-b_S_13-b_*) flowers at SL stigmas just start to open but were emasculated before anther dehiscence. Chemicals (cerulenin) treatment of stigmas followed the methods of the stigma feeding assays [56] with some modifications. Inserted in PGM containing the cerulenin and treated for 3 h and 6 h after pollination, the stigmas were subjected to aniline blue assay for observation of pollen grain attachment and pollen tube growth through microscopy.

## 5. Conclusions

Analysis of the transcriptome using Illumina RNA-seq and PacBio SMRT-seq allowed us to identify the novel genes and metabolism pathways involved in the SI response. The KEGG pathway analysis revealed a significant enrichment of 11 and 14 key enzyme genes for flavonoid and fatty acid biosynthesis, respectively, in the “incompatible” group. Cluster analysis and qRT-PCR validation indicated that the drastically reduced expression levels of key flavonoid and fatty acid enzyme genes at stigma maturity stage may be closely related to SI response. The compatible pollen grain attachment and pollen tube growth were drastically reduced when the stigmas were pretreated with a FASN inhibitor; it is indicated that fatty acid accumulation in mature stigmas may be essential for compatible pollination. These findings may provide valuable information for further developing some novel candidate genes and material metabolic pathways that participate in the SI response.

## Figures and Tables

**Table 1:** Statistics of DEGs.

DEG Set	DEG Number	Up-Regulated	Down-Regulated
SS vs. SM	4178	2166	2012
SS vs. SL	6349	3063	3286
SM vs. SL	5185	2870	2315
Total: 15,712			

## Supplementary Materials

The following supporting information can be downloaded at: https://www.mdpi.com/article/10.3390/ijms232113102/s1.

## Abbreviations

SI	Self-Incompatibility
CP	Compatible Pollination
BUSCO	Benchmarking Universal Single-Copy Orthologs
FLNC	Full-Length Non-Chimeric
CCS	Circular Consensus Sequencing
lncRNAs	Long Non-Coding RNAs
AS	Alternative Splicing
APA	Alternative Polyadenylation
SSR	Simple Sequence Repeat
ICE	Iterative Clustering for Error Correction
NR	NCBI Non-Redundant Protein Sequences
HQ	High Quality
LQ	Low Quality
ORF	Open Reading Frame
SMRT	Single-Molecule, Real-Time
FPKM	Fragment Per Kilobase of Transcript Per Million Fragments mapped
DEG	Differentially Expressed Gene
TF	Transcription Factor
WGCNA	Weighted Gene Co-expression Network Analysis
GO	Gene Ontology
KEGG	Kyoto Encyclopedia of Genes and Genomes
qRT-PCR	Real-Time Quantitative PCR
FASN	Fatty Acid Synthase
