# Supplementary material for: Fatty Acid Biosynthesis Pathways Are Downregulated during Stigma Development and Are Critical during Self-Incompatible Responses in Ornamental Kale
Source: Int J Mol Sci. 2022 Oct 28;23(21):13102. doi: 10.3390/ijms232113102 (PMC9656282; doi:10.3390/ijms232113102)
Supplement: Supplementary file 1 [file ijms-23-13102-s001.zip › Supplymentary Figures/Supplymentary Figures.pdf]

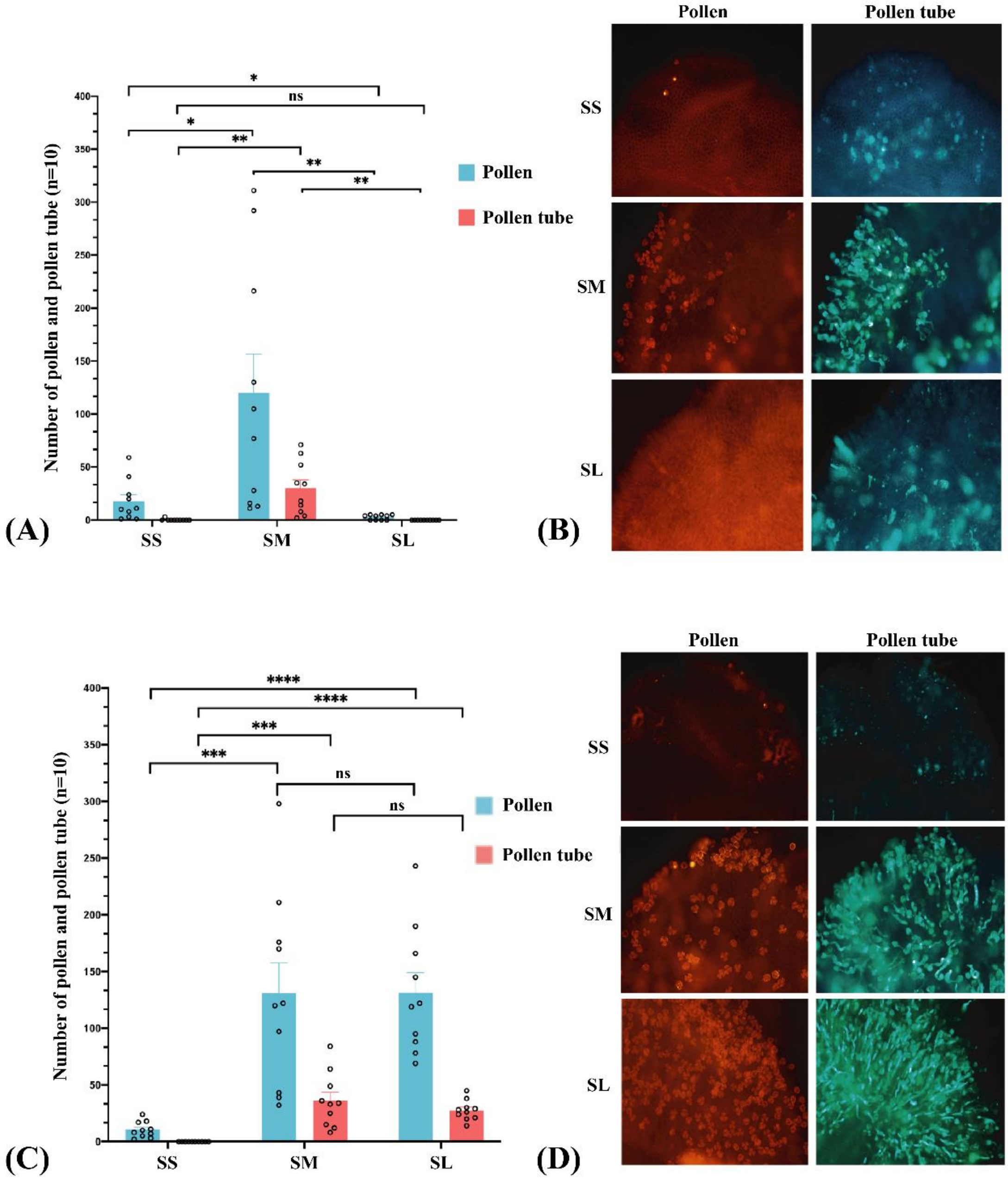

Figure S1. Observation of pollen and pollen tube number at stigma different developmental stages after self-incompatible (SI) and compatible pollination (CP). (A) Pollen and pollen tube number statistics. (**B)** Aniline blue staining of pollen and pollen tube after SI. **(C)** Pollen and pollen tube number statistics. (**D)** Aniline blue staining of pollen and pollen tube after CP. Asterisks indicate significant difference (*$p < 0.05$, **$p < 0.01$, ***$p < 0.001$, ****$p < 0.0001$ by Student's t-test), ns indicate no significant difference. Error bars represent the standard error of the mean (n=10). Scale bars=100μM.

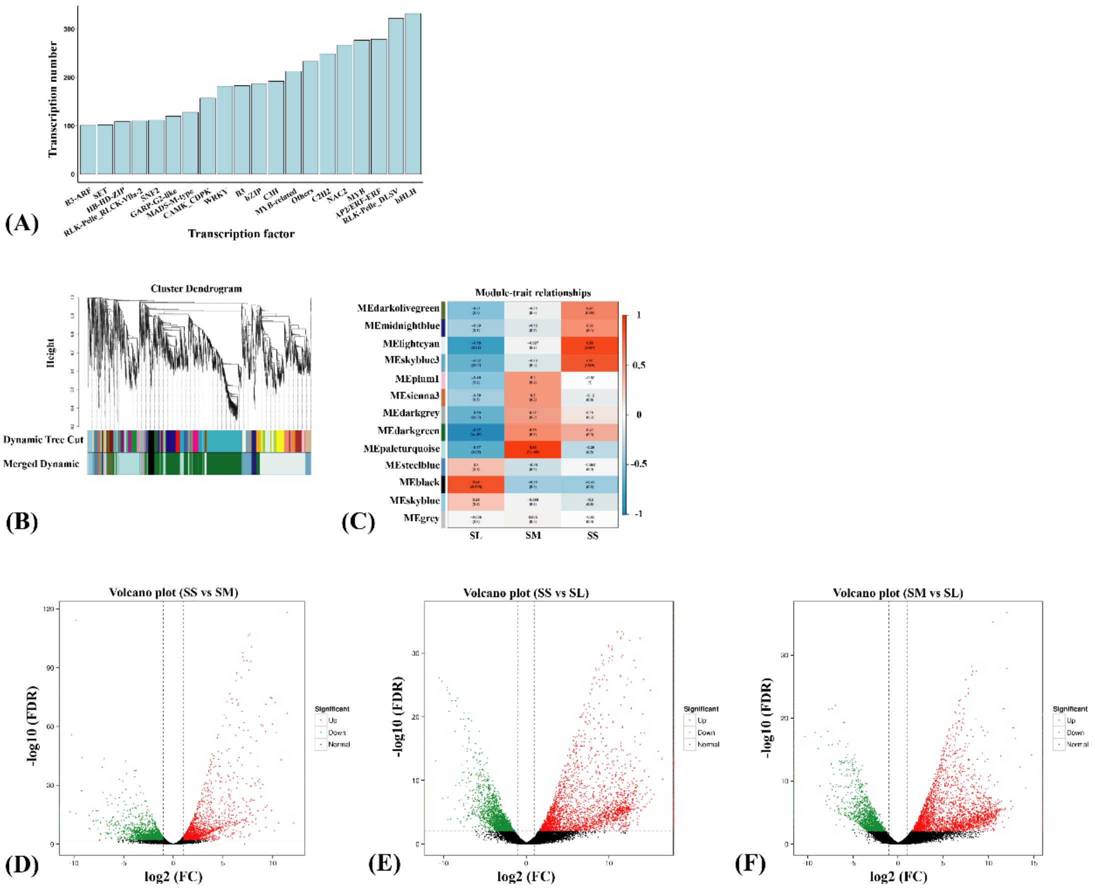

Figure S2. WGCNA analysis of TFs and prediction of DEGs. (A) Transcript number statistics of the top 20 TF families. **(B)** Hi-erarchical clustering tree of TFs by WGCNA. **(C)** Module-trait relationship analysis, the numbers inside the boxes were Pearson's correlation coefficient and their p-value in the brackets. Volcano plots show the statistics of DEGs in the three comparison groups **(D-F). (D)** SS vs SM. **(E)** SS vs SL. **(F)** SM vs SL.

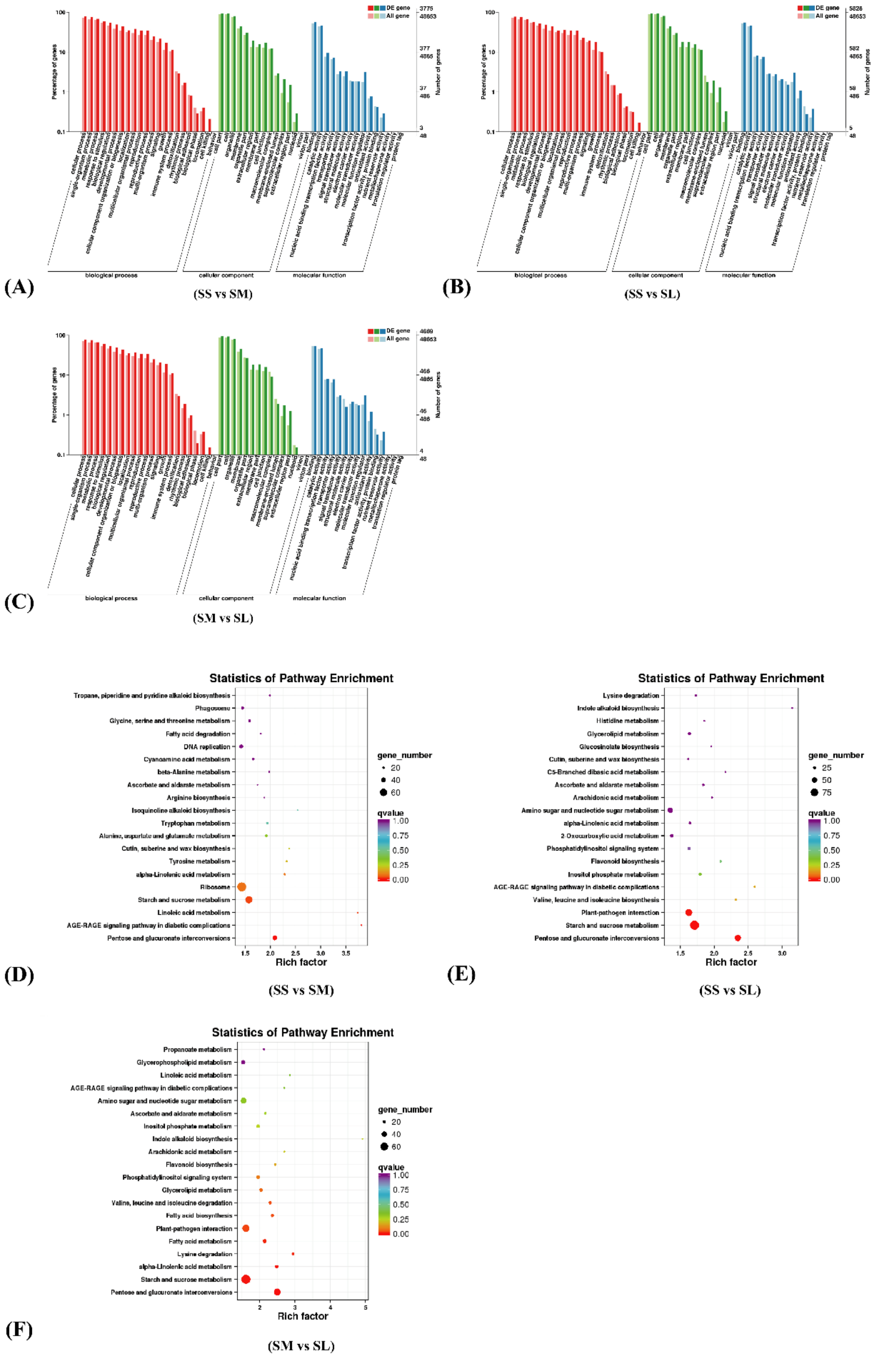

Figure S3. GO enrichment and KEGG enrichment statistics of DEGs in the three comparison groups. GO enrichment of DEGs **(A-C). (A)** SS vs SM. **(B)** SS vs SL. **(C)** SM vs SL. KEGG enrichment of DEGs **(D-F). (D)** SS vs SM. **(E)** SS vs SL. **(F)** SM vs SL.
